# Association of traditional cardiovascular risk factors with carotid atherosclerosis among adults at a teaching hospital in south-western Nigeria

**DOI:** 10.5830/CVJA-2018-014

**Published:** 2018

**Authors:** Dorcas Omisore Adeleye, Comfort Famurewa Olusola, Mopelola Asaleye Christiana, Adeyoyin Komolafe Morenikeji, Bimbola Fawale Michael, Ishmael Afolabi Babalola

**Affiliations:** Department of Radiology, Obafemi Awolowo University and Obafemi Awolowo University teaching hospitals complex, Ile-Ife, Nigeria Adeleye Dorcas Omisore, MB BS, FWACS, FMCR, omisoreadeleye@ yahoo.com; Department of Radiology, Obafemi Awolowo University and Obafemi Awolowo University teaching hospitals complex, Ile-Ife, Nigeria Adeleye Dorcas Omisore, MB BS, FWACS, FMCR, omisoreadeleye@ yahoo.com; Department of Radiology, Obafemi Awolowo University and Obafemi Awolowo University teaching hospitals complex, Ile-Ife, Nigeria Adeleye Dorcas Omisore, MB BS, FWACS, FMCR, omisoreadeleye@ yahoo.com; Department of Medicine, Obafemi Awolowo University, Ile-Ife, Nigeria; Department of Medicine, Obafemi Awolowo University, Ile-Ife, Nigeria; Department of Radiology, Obafemi Awolowo University teaching hospitals complex, Ile-Ife, Nigeria

**Keywords:** atherosclerosis, cardiovascular, carotid, Nigerian, risk factors

## Abstract

**Background:**

Traditional cardiovascular risk factors (CVRFs), which include age, gender, hypertension, diabetes mellitus, dyslipidaemia, smoking, alcohol consumption, chronic kidney disease and obesity, have been shown to be associated with atherosclerosis. We aimed to evaluate the impact of traditional CVRFs on carotid atherosclerosis (CA) in a sample of Nigerian adults.

**Methods:**

We examined 162 subjects with traditional CVRFs in a cross-sectional study. Demographic and clinical data, including history of hypertension, diabetes mellitus, smoking, alcohol intake and chronic kidney disease, as well as systolic and diastolic blood pressure, weight and height were collected. Serum creatinine, fasting blood glucose and lipid profiles were also determined. Carotid intima–media thickness (CIMT) and presence of carotid plaque (CP) were evaluated by high-frequency B-mode ultrasound. Chi-squared and regression analyses were carried out to determine associations between variables of CIMT and CVRF.

**Results:**

Increased CIMT was associated with all CVRFs (p < 0.05) except gender (p > 0.05), while CP was associated with older age, obesity, hypertension and dyslipidaemia (p < 0.05). We found prevalence of increased CIMT was 53.7%, while that of CP was 16.1%. The prevalence of CA (increased CIMT and CP) also increased with increasing number of CVRFs in the subjects. Age ≥ 50 years, hypertension, dyslipidaemia, obesity and alcohol intake explained 78.7% of variance in CIMT, while age ≥ 50 years and hypertension explained 38.0% of variance in CP.

**Conclusions:**

CA was associated with presence and increasing number of traditional CVRFs. A significant percentage of variance in CA was, however, unexplained by traditional CVRFs.

Atherosclerosis is the primary cause of morbidity and mortality in cardiovascular disease.^1^ It develops silently over decades, long before symptoms occur.^1^ Carotid intima–media thickness (CIMT) is a measure of subclinical atherosclerosis associated with cardiovascular risk factors (CVRFs) and is predictive of cardiovascular diseases such as myocardial infarction and stroke.^2^

Conventionally, CVRFs are divided into traditional and non-traditional factors.^3^ Traditional CVRFs, including age, gender, obesity, diet, hypertension, diabetes mellitus, dyslipidaemia, smoking, alcohol consumption and chronic kidney disease have been shown to account for most of the populationattributable risks for cardiovascular events.^3^-^5^ Existing evidence suggests an association between these individual traditional CVRFs and CIMT.^6^ Studies have gone further to establish the greater impact of multiple risk factors on CIMT compared with individual risk factors in different population groups.^6^,^7^ Traditional CVRFs, however, account for < 50% of the variance of atherosclerotic plaque burden and may not explain a large proportion of the variance in CIMT, especially when measured in plaque-free locations.^8^

Intima–media thickness is widely accepted as a valid marker for the process of atherosclerosis and has been applied in the study of cardiovascular disease for more than two decades.^1^ Carotid ultrasonography is a non-invasive means of identifying early-stage atherosclerotic changes in the arterial wall with the measurement of intima–media thickness and detection of plaque.^1^ Combined CIMT and plaque assessment is considered better than either measure alone in the assessment of atherosclerotic risk.^9^

Early detection of predictors of CIMT and their early modification may have a significant impact on the prevention of atherosclerotic disease.^1^ Therefore, to reduce the mortality rate associated with cardiovascular diseases such as stroke, it is clinically important to study the effects of CVRFs on carotid atherosclerosis. In this study, we aimed to evaluate the association of individual traditional CVRFs and the cumulative effect of multiple CVRFs with carotid atherosclerosis (CA) in a sample of Nigerian adults with established CVRFs.

## Methods

Adults aged 18 years and older were consecutively recruited from individuals who visited the medical out-patient, cardiology, neurology, hypertension and diabetes clinics of Obafemi Awolowo University teaching hospitals complex (OAUTHC), Ile-Ife, Nigeria, between January and December 2013. A total of 162 subjects were enrolled. Ethical approval was obtained from the Ethics and Research Committee of OAUTHC, Ile-Ife, and written informed consent was obtained from every study participant.

Demographic and clinical data such as age, gender, history of hypertension, diabetes mellitus, chronic kidney disease, smoking and alcohol intake were obtained by means of a structured data sheet. Blood pressure was measured in line with practice guidelines after 15 minutes of rest in the examination room, before ultrasound of the carotid artery was done. With patients seated comfortably, back supported, legs uncrossed, left upper arm bare and supported at heart level, an appropriate bladder cuff of an analogue mercury sphygmomanometer was applied to the left upper arm to encircled 80% or more of the arm circumference. After inflation, the mercury column was deflated at 2 to 3 mm/s. The first and last audible sounds were taken as systolic and diastolic blood pressure, respectively, and their measurements were given to the nearest 2 mmHg. Neither the patient nor the doctor taking the measurement talked during the procedure. Two readings at one-minute intervals were taken, and the average was recorded.

Weight and height were measured using a mechanical physician’s weighing scale attached to a stadiometer (model ZT-160, China). Body mass index (BMI) was calculated as weight (kilograms) divided by height (metres) squared.

Venous blood samples were taken from each participant between 07:00 and 08:00, after an overnight fast of eight hours. Samples were centrifuged within two hours of collection at 3 000 g for five minutes in a swing-bucket centrifuge, after which the serum was separated into plain plastic screw-capped containers and stored frozen at –20°C until analysis.

Samples were analysed in the chemical pathology department of the hospital. Plasma samples were analysed for glucose concentration (based on the glucose oxidase method) on the day of collection, while serum samples were analysed for other biochemical markers within one week of collection.

Creatinine (Cr) level was estimated in the serum with picric acid (Jaffe’s reaction); total cholesterol (TC) was determined with the cholesterol oxidase method; triglycerides (TG) were assayed with the glycerol phosphate oxidase/peroxidase method; and high-density lipoprotein cholesterol (HDL-C) was determined with the precipitation method. Assay kits for lipid profiles were purchased from Randox Laboratory Ltd, UK. Low-density lipoprotein cholesterol (LDL-C) was calculated using the empirical relationship of Friedewald’s formula:^10^

LDL = total cholesterol – HDL-C – TG/5

All components of the lipid profile are given in mmol/l. Normal values were taken as TC ≤ 5.2 mmol/l, HDL-C ≥ 1.03 mmol/l in men, ≥ 1.30 mmol/l in women, LDL-C ≤ 3.4 mmol/l, and TG ≤ 1.7 mmol/l, based on the National Cholesterol Education Program Adult Treatment Panel III (ATP III).^10^

Serum creatinine level is expressed in μmol/l, and values between 80 and 115 μmol/l in males and 53 and 97 μmol/l in females were considered normal.^10^ Fasting blood sugar levels of 4.1–5.6 mmol/l were considered normal.^10^ These laboratory assessments were done in collaboration with the chemical pathologists.

Definition of risk factors was guided by the ATP III guidelines.^10^ Hypertension was defined as resting systolic blood pressure (SBP) ≥ 140 mmHg, and/or diastolic blood pressure (DBP) ≥ 90 mmHg,^10^ or use of antihypertensive drugs. Dyslipidaemia was defined as use of antilipaemic drugs or having one or more of the following: TC ≥ 5.2 mmol/l, LDL-C ≥ 3.4 mmol/l, HDL-C ≤ 1.0 mmol/l, or TG ≥ 1.70 mmol/l.10 Diabetes mellitus (DM) was defined as fasting blood glucose (FBG) ≥ 7.0 mmol/l, or use of antidiabetes medication.^10^

A smoker was defined as a person who had smoked at least 100 cigarettes over his/her lifetime, including both current smoker (a person who continued to smoke daily or occasionally at the time of study) and past smoker (a person who had not smoked in the past 12 months).^11^ An alcohol consumer was defined as a person who imbibed alcohol, including current consumer (a person who had consumed alcohol in the past 12 months) and past consumer (a person who had consumed alcohol in the past, but not in the past 12 months).^11^

A history of peripheral artery disease, myocardial infarction, angioplasty, stroke or coronary artery bypass surgery was not recorded in our study participants.

Carotid ultrasonography was performed using a Mind-ray DC 7 ultrasound machine, equipped with a 7.5–12-MHz highresolution linear-array transducer. The common carotid artery was scanned for CIMT and measurements were taken 10 mm from the carotid bulb. Intima–media thickness was defined as the distance between the leading edge of the lumen–intima and the leading edge of the media–adventitia echo.^12^ An average of the right and left common carotid arteries (CCA) was taken for the study.

CA was defined as the presence of increased CIMT with or without carotid plaque (CP). CIMT ≥ 0.9 mm was taken as increased CIMT.^12^,^13^ Carotid plaques were recorded as present or absent if seen or not, respectively. Plaque was defined as focal thickening of at least 50% greater than that of the surrounding vessel wall, with a minimal thickness of at least 1.5 mm.^12^,^13^

## Statistical analysis

All analyses were performed using the Statistical Package for the Social Sciences (SPSS) statistical software (Version 20.0), SPSS Inc. Continuous variables are represented as mean ± standard deviation (SD) while categorical variables are represented as percentages. Group means of subjects with and without CVRFs were compared using the Student’s t-test, while proportions were compared using the chi-squared test. Bivariate logistic regression was used to compare associations of subjects with CVRFs with carotid atherosclerosis (increased CIMT or plaque presence) and those without (normal CIMT or plaque absence). Only variables with statistically significant associations on bivariate analysis were included in the final multivariate logistic regression model with the odds ratio and 95% CI presented. Significance was taken at p < 0.5.

## Results

Clinical, laboratory and anthropometric characteristics of the subjects are shown in Table 1. The mean age of the study participants was 51.96 ± 15.09 years and 49.4% were male. The mean values of Cr, BMI, SBP, DBP, TC, TG, HDL-C, LDL-C and prevalence of CVRFs are shown in Table 1. The prevalence of carotid plaque among the study participants was 16.1%, and 87 (53.7%) of the subjects had increased CIMT (≥ 0.9 mm).

**Table 1:** Clinical, laboratory and anthropometric characteristics of the study sample

*Variables*	*Statistics*
Age (years) (mean ± SD)	51.96 ± 15.09
BMI (kg/m^2^) (mean ± SD)	27.98 ± 5.59
Gender (male), n (%)	80 (49.4)
SBP (mmHg) (mean ± SD)	141.27 ± 29.95
DBP (mmHg) (mean ± SD)	89.49 ± 21.00
FBS (mmol/l) (median (Q1–Q3)	4.00 (3.80–5.53)
Cr (μmol/l) (mean ± SD)	100.13 ± 24.26
TC (mmol/l) (mean ± SD)	5.34 ± 1.25
TG (mmol/l) (median (Q1–Q3)	1.96 (1.50–3.61)
HDL-C (mmol/l) (mean ± SD)	1.23 ± 0.31
LDL-C (mmol/l) (mean ± SD)	3.59 ± 1.03
Age ≥ 50 years, n (%)	83 (51.2)
Hypertension, n (%)	80 (49.4)
Diabetes mellitus, n (%)	45 (27.8)
Dyslipidaemia, n (%)	111 (68.5)
Obesity, n (%)	56 (34.6)
Alcohol, n (%)	45 (27.8)
Smoking, n (%)	25 (15.4)
Chronic kidney disease n (%)	32 (19.8)
CIMT (mm) (median (Q1–Q3)	1.10 (0.70–1.40)
Increased CIMT, n (%)	87 (53.7)
Presence of plaque, n (%)	26 (16.1)

BMI: body mass index, SBP: systolic blood pressure, DBP: diastolic blood pressure, FBS: fasting blood sugar, Cr: serum creatinine, TC: serum total cholesterol, TG: serum triglycerides, HDL-C: serum high-density lipoprotein cholesterol, LDL-C: serum low-density lipoprotein cholesterol, CIMT: carotid intima–media thickness, SD: standard deviation, CVRFs: cardiovascular risk factors.

Mean values of CIMT were significantly increased with advancing age and in subjects with individual CVRFs compared with subjects without CVRFs (p < 0.05) (Table 2). CIMT was however not significantly different between male and female subjects. There was a significant association between increased CIMT and other traditional CVRFs (Table 3). However, age ≥ 50 years, hypertension, obesity, dyslipidaemia and alcohol intake remained independently associated with increased odds of increased CIMT (Table 3).

**Table 2:** Mean CIMT by CVRFs

*Risk factors*	*No of cases, total = 162*	*CIMT (mean ± SD) (mm)*	*p-value*
Age (years)
< 40	41	0.75 ± 0.23	< 0.001
41–50	42	1.08 ± 0.43
51–60	30	1.20 ± 0.34	
61–70	24	1.23 ± 0.33
> 70	25	1.10 ± 0.32
Gender
Male	80	1.12 ± 0.40	0.574
Female	82	1.08 ± 0.42
Smoking
Present	25	1.26 ± 0.30	0.038
Absent	137	1.07 ± 0.43
Hypertension
Present	80	1.34 ± 0.32	< 0.001
Absent	82	0.87 ± 0.36
Diabetes
Present	45	1.26 ± 0.36	0.003
Absent	116	1.04 ± 0.42
Dyslipidaemia
Present	111	1.23 ± 0.41	< 0.001
Absent	51	0.81 ± 0.24
Alcohol
Present	45	1.31 ± 0.37	< 0.001
Absent	117	1.02 ± 0.40
CKD
Present	130	1.25 ± 0.40	0.027
Absent	32	1.07 ± 0.41
Obesity
Present	56	1.34 ± 0.39	< 0.001
Absent	106	0.98 ± 0.37	

CIMT: carotid intima–media thickness, CVRFs: cardiovascular risk factors, CKD: chronic kidney disease.

**Table 3:** Association between risk factor variables and increased CIMT

**	*Normal CIMT *	*Increased CIMT*	*Bivariate model*			*Logistic regression model Nagelkerke R2 = 0.787*		
Variable	(%)	(%)	*UOR*	*95% CI*	*p-value*	*AOR*	*95% CI*	*p-value*
Age ≥ 50 years	21 (28.0)	62 (71.3)	6.38	3.2– 12.66	< 0.001	0.048	0.01–0.23	< 0.001
Gender (male)	36 (48.0)	44 (50.6)	1.11	0.598–2.056	0.744	NI	NI	NI
Obesity	10 (13.3)	46 (52.9)	7.293	3.317–16.032	< 0.001	0.163	0.03–0.77	0.022
Hypertension	10 (13.3)	70 (80.5)	26.77	11.43–62.68	< 0.001	0.035	0.008–0.149	< 0.001
Diabetes mellitus	10 (13.3)	36 (41.4)	4.59	2.08–10.12	< 0.001	0.20	0.037–1.07	0.060
Dyslipidaemia	32 (42.7)	79 (90.8)	13.27	5.62–31.32	< 0.001	0.03	0.02–0.428	0.009
Abnormal TC	15 (20.0)	48 (55.2)	4.92	2.43–9.98	< 0.001	0.99	0.17–5.60	0.987
Abnormal TG	23 (30.7)	66 (75.9)	7.11	3.55–14.23	< 0.001	11.68	1.12–122.1	0.040
Abnormal LDL-C	24 (32.0)	57 (65.5)	4.04	2.09–7.78	< 0.001	3.92	0.51–30.12	0.190
Abnormal HDL-C	3 (4.0)	17 (19.5)	5.83	1.64–20.77	0.003	0.83	0.11–6.39	0.859
Smoking	4 (5.3)	21 (24.1)	5.65	1.84–17.32	0.001	0.146	0.02–1.08	0.059
Alcohol	7 (9.3)	38 (43.7)	7.53	3.11–18.30	< 0.001	0.067	0.01–0.36	0.002
Chronic kidney disease	8 (10.7)	24 (27.6)	3.19	1.34–7.62	0.007	0.729	0.14–3.71	0.703

CIMT: carotid intima–media thickness, CI: confidence interval, TC: total cholesterol, TG: triglycerides, LDL-C: low-density lipoprotein cholesterol, HDL-C: highdensity lipoprotein cholesterol, UOR: unadjusted odds ratio, AOR: adjusted odds ratio.

**Fig 1:**
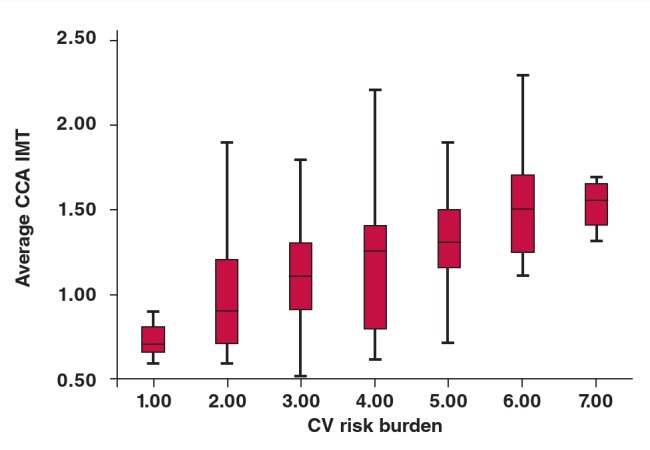
Box plots showing the relationship between CIMT and the number of CVRFs.

CP was associated with age ≥ 50 years (p < 0.001), obesity (p = 0.002), hypertension (p < 0.001) and dyslipidaemia (p = 0.001) (Table 4). Age ≥ 50 years and hypertension were the independent predictors of increased odds of CP (Table 4). Mean values of CIMT increased with increasing number of CVRFs, as shown in Fig 1.

**Table 4:** Association between risk factor variables and presence of plaques

**	*Plaque (–)*	*Plaque (+)*	*Bivariate model*			*Logistic regression model Nagelkerke R2 = 0.380*		
Variable	(%)	(%)	*UOR*	*95% CI*	*p-value*	*AOR*	*95% CI*	*p-value*
Age ≥ 50 years	61 (44.9)	22 (84.6)	6.76	2.21– 20.67	<0.001	0.21	0.06–0.73	0.014
Gender (male)	67 (49.3)	13 (50.0)	1.03	0.45–2.38	0.945	NI	NI	NI
Obesity	40 (29.4)	16 (61.5)	3.84	1.61–9.18	0.002	0.50	0.18–1.41	0.191
Hypertension	57 (41.9)	23 (88.5)	10.63	3.04–37.10	< 0.001	0.30	0.008–1.21	0.041
Diabetes mellitus	35 (25.7)	11 (42.3)	2.12	0.89–5.04	0.086	NI	NI	NI
Dyslipidaemia	86 (63.2)	25 (96.2)	14.54	1.92–110.55	0.001	0.18	0.01–2.81	0.220
Abnormal TC	44 (32.4)	19 (73.1)	5.68	2.22–14.50	< 0.001	0.22	0.05–1.08	0.063
Abnormal TG	67 (49.3)	22 (84.6)	5.66	1.85–17.30	0.001	1.42	0.27–7.60	0.681
Abnormal LDL-C	63 (46.3)	18 (69.2)	2.61	1.06–6.40	0.032	2.57	0.52–12.70	0.248
Abnormal HDL-C	13 (9.6)	7 (26.9)	3.49	1.23–9.84	0.014	0.64	0.19–2.13	0.468
Smoking	20 (14.7)	5 (19.2)	1.38	0.47–4.09	0.558	NI	NI	NI
Alcohol	34 (25.0)	11 (42.3)	2.20	0.92–5.25	0.071	NI	NI	NI
Chronic kidney disease	24 (17.6)	8 (30.8)	2.07	0.81–5.32	0.124	NI	NI	NI

Plaque (–): plaque absent, plaque (+): plaque present, CI: confidence interval, TC: total cholesterol, TG: triglyceride, LDL-C: low-density lipoprotein cholesterol, HDL-C: high-density lipoprotein cholesterol, UOR: unadjusted odds ratio, AOR: adjusted odds ratio.

## Discussion

The commonest traditional CVRFs besides age (51.2% of study sample were ≥ 50 years in age) in this study were dyslipidaemia (68.5%), hypertension (49.4%) and obesity (34.2%). Previous studies in Nigeria have sought to describe the prevalence of CVRFs in different populations, such as hypertensives, diabetics, and elderly and apparently healthy adult Nigerians.

Similar to our results, Akintunde et al.,^14^ in a cross-sectional study in the south-western region of Nigeria among apparently healthy university staff aged 27 to 73 years, with a mean age of 45.27 ± 7.87 years, found dyslipidaemia (49.5%), generalised obesity (44.7%) and hypertension (40.8%) to be the most prevalent CVRFs in their study population. Another populationbased cross-sectional study by Sani et al.^15^ in the north-western region of Nigeria among 300 apparently healthy Nigerians between 18 and 75 years, with mean age of 37.6 ± 10.6 years, found dyslipidaemia (28.3%), hypertension (25.7%) and generalised obesity (21.3%) most prevalent.

Despite our study being hospital-based among subjects with CVRFs, the commonest traditional risk factors in our study are similar to these two non-hospital-based studies among apparently healthy adult Nigerians. From these reports and ours, it appears that dyslipidaemia, hypertension and obesity rank highest in frequency among traditional CVRFs in Nigeria. This requires confirmation in larger population studies. The differences in CVRF frequencies between our study and other Nigerian studies may be due to age differences, lifestyle and environmental factors, as well as differences in clinical characteristics.

In our study, more than half of the subjects (53.7%) had increased CIMT (≥ 0.9 mm) and one in six (16.1%) had CP. Even though each of our study subjects had at least one CVRF, 46.3% of them still had CIMT within normal limits. A possible explanation for this finding is that the subjects with normal CIMT values probably had had risk factors for a shorter time, since CIMT has been shown to worsen with longer exposure to CVRFs.^16^

In a Nigerian study by Okeahialam et al.^17^ in 70 hypertensives, 70 diabetics and 71 non-diabetic non-hypertensive controls aged 30 years and older at a teaching hospital in north-central Nigeria, the prevalence of CA in their population was 47.5% for diabetics, 48.9% for hypertensives and 36.5% for the controls. They did not find any significant difference in CIMT between the subjects with hypertension and DM and the controls, although they found a reasonable degree of CA in their controls. Therefore they suggested that there is a need to evaluate for other traditional risk factors other than hypertension and DM, and for novel emerging risk factors.

In our cross-sectional study, we evaluated for more traditional risk factors than just hypertension and DM, and our subjects had at least one CVRF. Comparing our subjects to controls who had no traditional CVRFs may have shown significant differences in CIMT between the two groups, as has been demonstrated by other researchers.^6^,^7^

In the present study, CIMT increased with advancing age until the seventh decade, after which it decreased. However, the difference between CIMT in the seventh and eighth decades was not statistically significant on post hoc analysis (p > 0.05). Also, 70.^1^ and 84.6% of subjects with increased CIMT and CP, respectively, were ≥ 50 years of age. Ayoola et al.,^18^ in a previous study in the same location as ours in 200 hypertensives with a mean age of 58.8 ± 11.6 years and 100 controls with a mean age of 54.9 ± 10.9 years, found age to be an important predictor of CCA IMT in both groups of subjects. Similarly, Ibinaiye et al.^19^ found among hypertensives that CCA IMT increased with age from 21 to 70 years in a study population sample drawn from northern Nigeria. Ren et al.^6^ also showed that middle-aged and older adults with CVRFs displayed increased CIMT and higher grades of severity than the younger age groups in Chinese subjects.

We found a relationship between traditional CVRFs and CA in this study. Increased CIMT was independently predicted by age ≥ 50 years (six times the unadjusted odds and 0.05 times the adjusted odds in those under 50 years), hypertension (26 times the unadjusted odds and 0.04 times the adjusted odds in those without hypertension), intake of > 2 g/day of alcohol (7.5 times the unadjusted odds and 0.07 times the adjusted odds in those who had never drunk or not taken in the past year), obesity (seven times the unadjusted odds and 0.2 times the adjusted odds in the non-obese) and dyslipidaemia (13 times the unadjusted odds and 0.03 times the adjusted odds in the non-obese).

A Brazilian study by Baroncici et al.^20^ among 533 CVRF subjects with a mean age of 67.06 ± 12.44 years found male gender in addition to hypertension and age to be risk factors that increased CIMT. Gender was however not associated with increased CIMT in our study. The differences in CVRFs associated with CA in these studies could be due to ethnoracial differences, which should be confirmed in larger multiracial studies.

Another key finding of this study was that CIMT increased as the number or burden of CVRFs increased. Clustering of CVRFs was seen in our sample population and the value of CIMT paralleled the number of CVRFs in a linear, dose-dependent fashion. The risk of atherosclerosis increases with increasing burden of CVRFs. Previous studies^6^,^21^,^22^ have confirmed the greater impact of multiple risk factors on CIMT than individual CVRFs in different population groups despite differences in age, number of risk factors and race of the subjects and the carotid segments studied.

The prevalence of CP in this study was 16.1%. The prevalence of CP reported from other studies varies quite widely, despite comparability in sonographic methods. This may be explained by differences in sample characteristics, prominent among which are racial and environmental differences. Umeh et al.,^13^ in a study of normotensive and hypertensive subjects, found an overall prevalence of CP of 25.8% (29.2 and 22.5% in the hypertensive and normotensive subjects, respectively), which is higher than our prevalence despite similarities in the segment of carotid vessel where CIMT and CP were measured and the location of the studies. The older age of their sample compared to ours may partly explain the differences in CP prevalence. Interestingly, we found in our study that the presence of carotid plaque was independently predicted by age ≥ 50 years (seven times the unadjusted odds and 0.2 times the adjusted odds in those < 50 years) and hypertension (11 times the unadjusted odds and 0.3 times the adjusted odds in people without hypertension).

In support of our finding, a study in northern Nigeria,^19^ which measured CIMT and CP in the CCA, similar to our study, but in a slightly younger sample of hypertensive patients (mean age of 50.62 ± 10.46 years) than ours, expectedly found a lower CP prevalence of 10%. A hospital-based study similar to ours and the other two Nigerian studies,^13^,^19^ done among Brazilians,20 with a mean age of 67.06 ± 12.44 years, found the prevalence of CP to be 23.8% in their study, which is not unexpected, as the mean age of the subjects in their study was higher than in the three Nigerian studies.

Much higher prevalence of CP has been reported in the literature in population-based studies such as the Northern Manhattan Cohort Study (NOMAS),^8^ with a unique race/ ethnic distribution of community residents aged ≥ 39 years, which reported CP prevalence of 58% overall, 70% in Caucasian participants, 52% in Hispanics and 58% in blacks. Also, in Beijing, China,^23^ the prevalence of CP was 60.3% among urban residents aged 43–81 years, almost 70% in subjects ≥ 60 years, and 80% in those ≥ 70 years. The population-based study setting, which would have eliminated selection bias, in addition to the lower age range of the participants in our study (23–81 years) and the other three hospital-based studies by Umeh et al.,^13^ Ibinaiye et al.^19^ and Baroncici et al.,^20^ compared to the NOMAS (65–74 years) and Beijing studies (≥ 75 years) might explain the lower prevalence of CP found in our study and the three hospital-based studies.

From our study, age ≥ 50 years, hypertension, dyslipidaemia, obesity and alcohol intake > 20 g/day explained 78.7% of the variance in CIMT, while age ≥ 50 years and hypertension explained 38.0% of the variance in CP. This finding suggests that CIMT and CP may be influenced by different CVRFs, although age and hypertension influenced both. The relationships between CVRFs and carotid atherosclerosis could be properly evaluated in a longitudinal study.

It is not surprising that age and hypertension rank high in the prediction of CIMT and CP because they happen to be the most important risk factors for stroke. Age is the most important non-modifiable risk factor for stroke, while hypertension is the most important modifiable risk factor. Santos et al.,^24^ in a multicentre Brazilian study, found traditional CVRFs explained 14.1 to 37.3% of the CIMT variance. Kuo et al.,^8^ in the NOMAS study, found age, SBP, DBP, blood pressure- and lipid-lowering medications and diabetes to be the traditional risk factors that predicted CP and they explained 19.5% of variance in CP burden.

This difference can most likely be attributed to different characteristics of the study populations (race, age), the carotid segments measured, and study designs. Kuo et al.^8^ measured near and far walls of the CCA, the bulb and internal carotid artery (ICA) on both sides, while Santos et al.^24^ measured the far wall of the CCA, similar to us. Our study was a hospital-based study, in contrast to the population-based studies by Kuo et al.^8^ and Santos et al.^24^ Another hospital-based study like ours found age, gender, pack-years of smoking, SBP, DBP, DM, HDL-C, and blood pressure- and lipid-lowering medications to be the most significant determinants of carotid plaque area, explaining 52% of the variance in total plaque area (TPA).^25^ Apart from the difference in the predictors of CP between our study and this hospital-based study, the difference in the measurement of plaque considered (plaque thickness in our study versus total plaque area in theirs) may explain the higher percentage of variance in CP in that study.

## Limitations

The evidence from this study is limited by its cross-sectional design and hospital-based setting. The analysis was also limited to the CCA, which might not have detected the presence of atherosclerosis in other vascular beds or the more distal segments of the carotid artery. However, while prospective studies, which will generate IMT data in the carotid bulb and ICA are pending, our findings provide important insights into the determinants of subclinical CA in this population. Most of the subjects in the present study presented with more than one risk factor and received more than one medical therapy. However, assessment of the effects of each medication on each risk factor, as well as the effects of the prescribed medication on CIMT was not performed.

## Conclusion

This study provides evidence that a linear, graded and independent association exists between CVRFs and CIMT and that the risk of CP increases with increasing number of CVRFs. Age ≥ 50 years, hypertension, obesity and alcohol intake > 20 g/day were independent contributors to CIMT variance, while age ≥ 50 years and hypertension were the contributors to CP variance. About 21.3% of CCA IMT and 62.0% of CP could not be explained by traditional CVRFs in this study. This observation represents strong evidence to encourage future studies focusing on the influence of novel CVRFs on CIMT and CP.
